# Psychosocial burden of cardiac patients: a retrospective in-patient analysis

**DOI:** 10.1186/s12872-026-05761-5

**Published:** 2026-03-21

**Authors:** Katharina von Westerholt, Panagiotis Xynogalos, Norbert Frey, Hans-Christoph Friederich, Jobst-Hendrik Schultz, Bastian Bruns

**Affiliations:** 1 Department of General Internal Medicine, Psychosomatics, and Psychotherapy, Heidelberg University Hospital, Heidelberg, Germany; 2 Department of Cardiology, Angiology and Pneumology, Heidelberg University Hospital, Heidelberg, Germany; 3 DZHK (German Centre for Cardiovascular Research), Partner Site, Heidelberg/Mannheim, Heidelberg, Germany

**Keywords:** Heart failure, Depression, Anxiety, Diabetes, ICD, Cardiac decompensation

## Abstract

**Background:**

Cardiac patients face a higher risk of developing depression or anxiety, displaying a bidirectional risk-relationship. While previous studies have focused on specific cardiac subgroups, such as heart failure (HF) populations, our study investigates a broad cardiac population to explore cofactors most strongly correlating with depressive or anxious symptoms.

**Methods:**

A total of *n* = 511 patients (mean age 63.57 ± 15.86 years, 41.9% female, 58.1% male) with heterogenous admission diagnoses admitted to the “Siebeck” ward of the Centre of Internal Medicine at the University Hospital Heidelberg were retrospectively analyzed. Psychiatric symptoms were quantified using the Patient Health Questionnaire-9-item depression module (PHQ-9), the 7-item Generalized Anxiety Disorder scale (GAD-7) and the Short Form-36 (SF-36) to evaluate health-related quality of life (HRQoL). Regression analyses included the variables sex, age, HF (mild, moderate and severe), the presence of an implanted cardioverter defibrillator (ICD), former or current cardiac decompensation (CD), cardiomyopathy (CM), and diabetes mellitus (DM) as predictors. A *p* < 0.05 was considered statistically significant.

**Results:**

Younger age (PHQ-9: β = -0.117, *p *= 0.012; GAD-7: β = -0.205, *p* < 0.001) and female sex (PHQ-9: β = 0.118, *p* = 0.01; GAD-7: β = 0.164, *p* < 0.001) were significantly associated with elevated PHQ-9 and GAD-7 scores. CD (β = 0.149, *p* = 0.001), an ICD (β = 0.15, *p* = 0.002), and comorbid DM (β = 0.095, *p* = 0.036) were significantly associated with higher depression scores, while an ICD additionally correlated with more anxiety (β = 0.112, *p* = 0.024). Lower physical HRQoL was present in patients with DM (β = -0.163, *p* = 0.001) and CD (β = -0.147, *p* = 0.004). Relatively low adjusted R² values in the regression models reflect that only a small proportion of the variance in PHQ-9 (R² = 0.094) and GAD-7 (R² = 0.079) can be explained. Although severe HF was associated with higher PHQ-9 scores, the multivariate regression analyses did not confirm a significant association.

**Conclusions:**

Depressive and anxious symptoms, as well as physical HRQoL, are more strongly linked to specific clinical characteristics than to HF severity itself. Instead, younger age, female sex, CD, an ICD, and comorbid DM showed stronger associations with significantly increased depressive symptoms and lower HRQoL, while those with an ICD additionally described higher levels of anxiety. These findings support targeted psychosocial screening in these high-risk cardiac subgroups.

## Background

Heart diseases are among the leading causes of morbidity and mortality worldwide [1, 2]. Beyond their somatic burden, they are also associated with a two to three times higher prevalence of depressive and anxious symptoms as well as significantly reduced Health related quality of life (HRQoL) compared with the general population [3–5]. Cardiac patients with depressive or anxious symptoms are at an increased risk for adverse clinical outcomes such as increased morbidity, higher health care costs, elevated risk of rehospitalization and mortality [6].

Most existing studies examine psychological symptoms within specific subgroups, particularly heart failure (HF), while in clinical practice the associations are complex and cardiac inpatients often present with multimorbidity. In 2019, a systematic review published in “The Lancet Psychiatry” estimated the global prevalence of depressive disorders at 3.8% [7], while recent meta-analyses observed substantially higher prevalences of depression with 24.7% in individuals with HF and 19.8% in those with coronary heart disease (CHD) [8]. For anxiety the global prevalence is comparable to depression with an estimated 301.4 million individuals worldwide (3.8% of the population) affected, while among HF and CHD patients the prevalence of anxiety is substantially higher, ranging from 20% to 50% across different studies [9, 10]. Comparable elevated rates of depression and anxiety have been reported in other cardiac diseases [3, 4, 11, 12]. Further, individuals with atrial fibrillation or with an implantable cardioverter-defibrillator (ICD) also demonstrate an increased risk for depressive symptoms [13, 14] as well as non-cardiac comorbidities. For example, diabetes mellitus (DM) is also known to be associated with an increased risk for depression [15, 16]. Conversely, patients with preexisting depression or anxiety have a 1.2-fold increased risk of developing a heart disease compared to those without, underlining a bidirectional risk [6, 17, 18]. Due to the overlap of psychosomatic and cardiac symptoms, depression and anxiety are often overlooked by medical physicians, and both depressive and anxious symptoms are often difficult to clearly separate, since they positively correlate with each other [10, 19].

Pinpointing risk factors for mental health disorders in cardiac patients is essential for developing targeted approaches to improve patient outcome, quality of life and minimize health care burden [20].

The primary goal of this study was to assess the psychosocial burden in a heterogeneous cardiac inpatient population. The secondary objectives were to identify clinical characteristics and comorbidities - such as HF, CHD, cardiac decompensation (CD), DM or an ICD - that are most strongly associated with depressive or anxious symptoms.

## Methods

### Study design and patient population

A total of *n* = 511 Patients were included in the retrospective study. All patients were admitted to the “Siebeck” ward of the Centre of Internal Medicine at the University Hospital Heidelberg between January 2009 and July 2020. Four standardized outcome scores were predefined and calculated from the admission questionnaires: the Patient Health Questionnaire-9-item depression module (PHQ-9), the 7-item Generalized Anxiety Disorder scale (GAD-7), the physical- (PCS) and the mental component summary scale (MCS). Patients were included if they completed a sufficient proportion of the admission assessment to allow calculation of at least one valid outcome score. Score calculation was conducted according to the official recommendations of scoring for each score, respectively. Only data of patients aged 18 years or older at the time of admission, who completed sufficient portions of the admission assessment to meet the predefined criteria for calculating at least one valid outcome score of the four standardized scores mentioned below, were included. No other exclusion criteria were used. The study protocol fulfilled the Declaration of Helsinki. The Ethics Committee of the University Heidelberg waived the need for informed consent (S-210/2023), as obtaining consent from all included patients would have involved disproportionate effort and involved no disadvantages for the patients regarding their medical treatment or outcome. For the investigation only preexisting data was used without any direct involvement in patient’s treatment or procedures.

### Measurement

The admission form contained socio-demographic information such as age and gender as well as two standardized international psychological tests in German language. The questionnaires were assessed during acute hospitalization as part of routine clinical assessment and were used as screening instruments to assess the severity of depressive and anxiety symptoms. These measures were not intended to establish psychiatric diagnoses. Since the questionnaires were used not only for this study but also for routine clinical purposes,they were employed in their German versions. Psychiatric symptoms were quantified using the German Patient Health Questionnaire (PHQ-D) [21] consisting of the two standardized psychometric questionnaires PHQ-9, and the GAD-7 [21]. The PHQ-D was translated in the state-of-art procedures for test translation [22], and numerous studies have shown that the PHQ-9 and the GAD-7 are reliable and valid instruments for assessing depressive and anxious symptoms and both have been shown to have a high internal consistency [23–25]. Additionally, the patient’s HRQoL was assessed with the German version of the Short Form-36 (SF-36) [26], which is the most used health-related measurement of quality of life [27]. The German version has been found to be a valuable tool in clinical studies [28]. Its results can be quantified by the PCS and MCS.

HF patients were categorized by their left ventricular ejection fraction (LVEF) in mild (> 40%), moderate (30–40%) and severe (< 30%) HF, with moderate and severe HF corresponding to heart failure with reduced ejection fraction (HFrEF), which is defined by the current European Society of Cardiology (ESC) classification by a LVEF ≤ 40%.

### Statistical analysis

For the comparison of continuous variables between different subgroups independent t-tests were used and presented as mean (M) ± standard deviation (SD). Further multivariate regression analyses were performed to control for different variables. The following independent variables were used as predictors in our models: age, sex, mild, moderate and severe HF, an ICD, cardiac CD, cardiomyopathy (CM) and DM. For statistical analysis IBM SPSS (Version 29) was used and a *p* < 0.05 was considered statistically significant.

## Results

### Descriptive statistics

A descriptive analysis of the data is presented in Table 1. *N* = 511 patients had a mean age of 63.57 years ± 15.86 with an age range from 18 to 97 years. 41.9% (*n* = 214) were female and 58.1% (*n* = 297) male. Patients presented with heterogeneous admission diagnoses: with 49.9% (*n* = 255) of all patients having a diagnosed HF, among whom 14.5% (*n* = 74) presented with a mild, 16.8% (*n* = 86) with a moderate and 18.6% (*n* = 95) with severely impaired LVEF. Further, 11.5% (*n* = 59) had a current or past CD and 9% (*n* = 46) had an ICD. Components of the metabolic syndrome were common, including DM with 30.7% (*n* = 157) of patients suffering from it.

**Table 1:** Baseline demographic and clinical characteristics of study participants

Variable	*n*	%	M	SD
Age (in years)			63.57	15.86
Female Sex	214	41.9		
Male Sex	297	58.1		
German Nationality (yes)	436	85.3		
Living with others	366	71.6		
Married	292	57.1		
Higher Education	102	23		
Retired	297	62.5		
HF	255	49.9		
>40% EF	74	14.5		
30-40% EF	86	16.8		
≤30% EF	95	18.6		
ICD	46	9.0		
CD	59	11.5		
CM	82	16		
CHD	359	70.3		
Myocardial Infarction	157	30.7		
Atrial Fibrillation	131	25.6		
COPD	72	14		
CABG	71	13.9		
DM	157	30.7		
Arterial Hypertension	391	76.5		
Hypercholesterinemia	219	42.9		
Obesity	141	27.6		
Current Psychopharmacological Treatment	118	23.1		
Psychotherapy (Current or Past)	126	24.6		
Length of hospital stay (in days)			7.81	12.80
PHQ-9 (*n*=476)			7.35	6.09
GAD-7 (*n*=474)			5.15	5.28
MCS (SF-36) (*n*=380)			46.02	12.55
PCS (SF-36) (*n*=380)			34.78	12.11

Total of *n*=511 patients. Categorial Variables are presented with absolute numbers (*n*) and percentage (%), metrical variables with mean (M) and standard deviation (SD). *HF* heart failure, *EF* ejection fraction, *CD* cardiac decompensation, *ICD* implantable cardioverter defibrillator, *CHD* coronary heart disease, *COPD* chronic obstructive pulmonary disease, *CABG* coronary artery bypass grafting, *CM* cardiomyopathy, *DM* diabetes mellitus, *PHQ-9* 9-item Patient Health Questionnaire- depression module, *GAD-7 *7-item Generalized Anxiety Disorder scale,*SF-36* 36-item Short Form Health Survey,*MCS* mental component scale (SF-36),*PCS* physical component scale (SF-36)

Regarding the psychological self-assessment scores, the average GAD-7, which ranges from 0 to 21 with higher scores suggesting a greater likelihood of clinically relevant anxious symptoms, was 5.15 ± 5.28 among the *n* = 474 patients with a valid sum score. For the PHQ-9 depression score, ranging from 0 to 27 with higher scores suggesting a higher burden of depression, *n* = 476 patients filled out the questionnaire sufficiently with an average score of 7.35 ± 6.09. For the SF-36, the MCS and PCS scores are norm-based ranging from 0 to 100 with a population mean of 50 and a standard deviation of 10 with higher scores suggesting a better quality of life. In our sample, the *n* = 380 patients presented a mean MCS of 46.02 ± 12.55, while the average PCS was 34.78 ± 12.11.

### Cofactors correlating with higher anxiety and depression scores

**Fig. 1:**
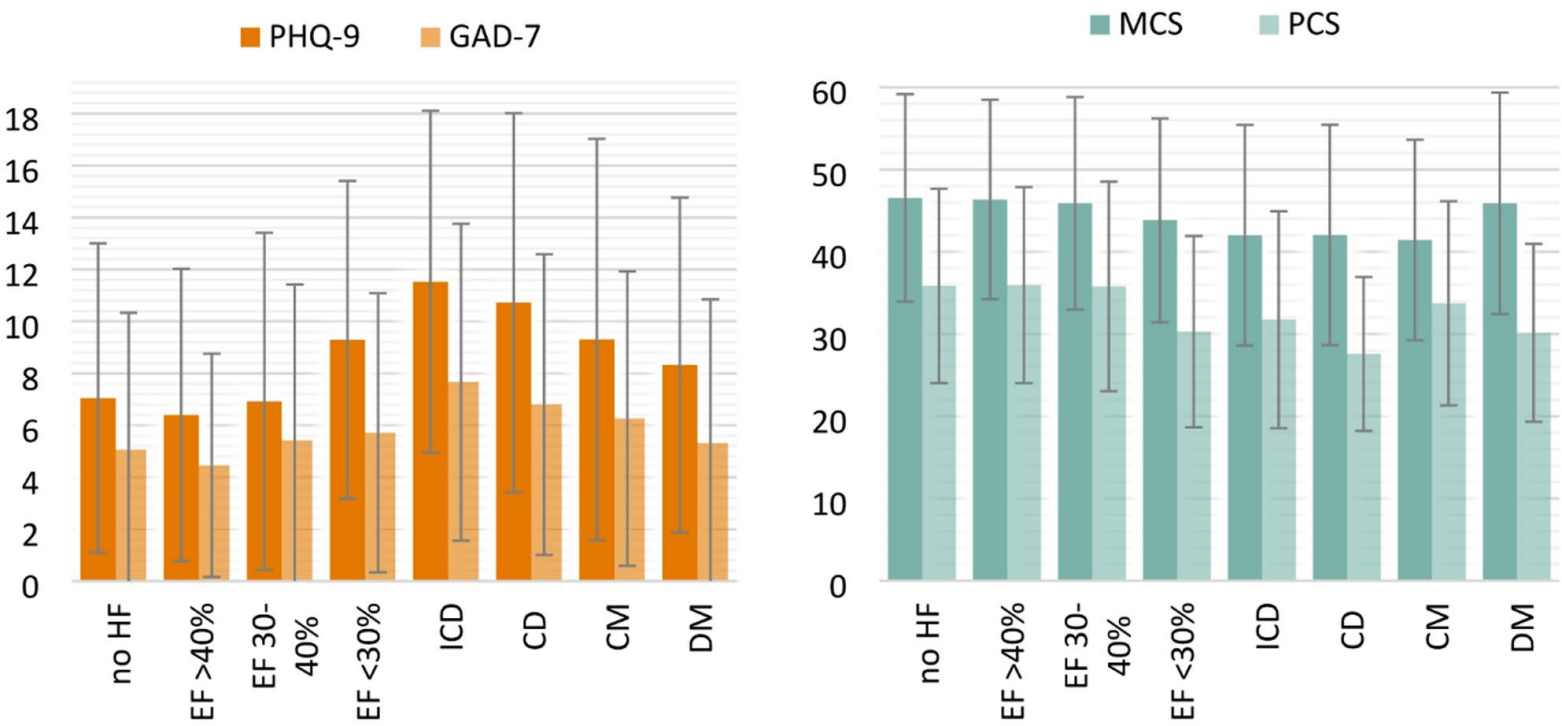
PHQ-9, GAD-7, and SF-36 scores of corresponding subgroups. Orange figure: mean PHQ‑9 depression scores and GAD‑7 anxiety scores for patients in the listed subgroups. Blue figure: mean Short Form‑36 (SF‑36) MCS and PCS scores for the same subgroups. Bars represent group means and error bars represent standard deviation (SD). HF=heart failure, EF=ejection fraction, ICD=implantable cardioverter defibrillator, CD=cardiac decompensation, CM=cardiomyopathy, DM=diabetes mellitus, PHQ-9 = 9-item Patient Health Questionnaire- depression module, GAD-7 = 7-item Generalized Anxiety Disorder scale, MCS=mental component scale, PCS =physical component scale.

Table 2 shows an overview of the average PHQ-D and SF-36 scores for different subgroups that were defined by different HF severity and comorbidities. Patients with severely impaired LVEF (<30%) had a significantly higher mean PHQ-9 of 9.29 ± 6.12, *p*=0.001 compared to all other patients. Patients with mild or moderate heart failure did not exhibit significantly elevated PHQ-9 scores, with an average score of 6.4 ± 5.63 for those with a LVEF ≥40% and 6.92 ±6.49 for those with a LVEF of 30-40%. Patients without HF (7.05 ± 5.96) scored lower than those with HF (7.66 ± 6.22) in irrespective of LVEF. Further, patients with an ICD had the highest depression scores with 11.53 ± 6.58, *p*<0.001 and the highest anxiety scores with an average of 7.66 ± 6.1, *p*=0.006. Those patients suffering from a CM (9.3 ± 6.73, *p*=0.002) and a DM (8.32 ± 6.45, *p*=0.022) also presented higher depression scores. Another important cofactor of HF patients regarding psychological comorbidities is CD, which correlated with elevated scores of all questionnaires assessed, with decompensated patients presenting higher depression (10.72 ± 7.3, <0.001) and anxiety scores (6.8 ± 5.79, *p*=0.016) as well as lower quality of life in both the MCS (42.04 ± 13.39, *p*=0.023) and PCS (27.58 ± 9.36, *p*<0.001) compared to all other patients. For the physical component of the SF-36 (PCS), patients with severe HF (30.28 ± 11.61, *p*<0.001) and those with DM (30.14 ± 10.81, *p*<0.001) scored significantly lower than other patients. In contrast, for the mental component of the SF-36 (MCS), patients with a CM (41.41 ± 12.19, *p*=0.002) had lower scores, while the presence of an ICD showed a trend towards significance (42 ± 13.41, *p*=0.050) (Fig. 1).

**Table 2:** Tabular overview of PHQ-9, GAD-7, and SF-36 scores for different subgroups

Variable	PHQ-9 (*n* = 477)		GAD-7 (*n* = 474)		MCS (*n* = 380)		PCS (*n* = 380)	
	M (SD)	*p*-value	M (SD)	*p*-value	M (SD)	*p*-value	M (SD)	*p*-value
No HF	7.05 (5.96)		5.06 (5.27)		46.53 (12.62)		35.83 (11.82)	
HF	7.66 (6.22)	0.279	5.25 (5.31)	0.702	45.53 (12.5)	0.439	33.77 (12.33)	0.099
>40% EF	6.4 (5.63)	0.174	4.46 (4.3)	0.169	46.35 (12.12)	0.831	35.93 (11.93)	0.442
30–40% EF	6.92 (6.49)	0.493	5.42 (6)	0.632	46.71 (12.92)	0.626	35.78 (12.73)	0.463
≤30% EF	9.29 (6.12)	0.001*	5.71 (5.37)	0.271	43.82 (12.39)	0.099	30.28 (11.61)	< 0.001*
ICD	11.53 (6.58)	< 0.001*	7.66 (6.1)	0.006*	42 (13.41)	0.050*	31.73 (13.19)	0.124
CD	10.72 (7.3)	< 0.001*	6.8 (5.79)	0.016*	42.04 (13.39)	0.023*	27.58 (9.36)	< 0.001*
CM	9.3 (6.73)	0.002*	6.26 (5.67)	0.044*	41.41 (12.19)	0.002*	33.73 (12.41)	0.460
DM	8.32 (6.45)	0.022*	5.32 (5.53)	0.656	45.89 (13.47)	0.9	30.14 (10.81)	< 0.001*

Comparison between corresponding subgroups and all other patients. *M* mean, *SD* standard deviation, *HF* heart failure, *ICD* implantable cardioverter defibrillator. *CD* cardiac decompensation, *CM* cardiomyopathy, *DM* diabetes mellitus, *PHQ-9* 9-item Patient Health Questionnaire depression module, *GAD-7* 7-item Generalized Anxiety Disorder scale, *MCS* mental component scale (SF-36), *PCS* physical component scale (SF-36)

*Marks significant (*p*<0.05) *p*-values by Student’s T-test of subgroup vs. all other patients

### Multivariate regression

To control for different subgroups a multivariate regression was performed (Table 3). The regression model included age, sex, the cofactors ICD, CD, CM and DM as well as HF grouped by LVEF of ≥ 40%, 30–40% and < 30% and was performed for all four psychosocial scores. For the PHQ-9 the model was significant [adjusted R² = 0.094, F (9, 466) = 6.464, *p* < 0.001)] with younger age (β = -0.117, *p* = 0.012), female sex (β = 0.118, *p* = 0.01), ICD (β = 0.15, *p* = 0.002), CD (β = 0.149, *p* = 0.001), and DM (β = 0.095, *p* = 0.036) being significant predictors for higher depression scores. While patients with severe HF vs. no HF presented with a higher mean PHQ-9 score (9.29 ± 6.12 vs. 7.05 ± 5.96), this difference was not significant after adjustment for confounders in multivariate regression analyses.

**Table 3:** Multivariate regression of PHQ-9, GAD-7, and SF-36 scores

Variables	PHQ-9			GAD-7			MCS			PCS		
	Std. β	B	*p*-value	Std. β	B	*p*-value	Std. β	B	*p*-value	Std. β	B	*p*-value
Age	-0.117	-0.044	0.012*	-0.205	-0.068	<0.001*	0.119	0.092	0.029*	-0.201	-0.15	<0.001*
Female sex	0.118	1.458	0.01*	0.164	1.755	<0.001*	-0.09	-2.298	0.087	-0.138	-3.412	0.005*
Mild HF	-0.019	-0.34	0.682	0.01	0.156	0.827	-0.014	-0.493	0.8	0.047	1.594	0.369
Moderate HF	0.001	0.018	0.981	0.059	0.85	0.217	-0.002	-0.06	0.974	0.009	0.275	0.87
Severe HF	0.083	1.307	0.131	0.052	0.699	0.355	-0.056	-1.803	0.366	-0.111	-3.426	0.06
ICD	0.15	3.194	0.002*	0.112	2.041	0.024*	-0.017	-0.744	0.77	-0.052	-2.219	0.339
CD	0.149	2.827	0.001*	0.081	1.356	0.081	-0.118	-3.513	0.088	-0.147	-5.474	0.004*
CM	0.015	0.244	0.772	0.011	0.153	0.835	-0.118	-4.023	0.087	0.05	1.66	0.362
DM	0.095	1.262	0.036*	0.044	0.507	0.336	0.0	-0.012	0.993	-0.163	-4.357	0.001*

Results of four separate multivariate regression analyses with the sum scores of PHQ-9, GAD-7, MCS, PCS as the dependent variables and the listed independent variables. Std. β Standardized β coefficient, *B* unstandardized coefficient, *HF* heart failure, *ICD* implantable cardioverter defibrillator, *CD* cardiac decompensation, *CM* cardiomyopathy, *DM* diabetes mellitus, *PHQ-9* Patient Health Questionnaire-9-item depression module, *GAD-7* 7-item Generalized Anxiety Disorder scale, *MCS* mental component scale (SF-36), *PCS* physical component scale (SF-36)

*p<0.05 was considered statistically significant

For the GAD-7 the regression model was significant [adjusted R² = 0.079, F (9, 464) = 5.509, *p* < 0.001)] with younger age (β = -0.205, *p* < 0.001), female sex (β = 0.164, *p* < 0.001), and ICD (β = 0.112, *p* = 0.024) being significant predictors for higher anxiety scores, while in contrast to depression CD or DM did not correlate with higher anxiety levels.

Regression models for the SF-36 were also significant for both the PCS [adjusted R² = 0.14, F (9, 368) = 7.792, *p* < 0.001)] and the MCS [adjusted R² = 0.034, F (9, 368) = 2.477, *p* = 0.009)]. While older age (β = -0.201, *p* < 0.001) and the female sex (β = -0.138, *p* = 0.005) suggested lower quality of life regarding the physical scale (PCS), the regression for the MCS showed that a younger age (β = 0.119, *p* = 0.029) was associated with worse mental quality of life. Regarding comorbidities a diagnosed CD (β = -0.147, *p* = 0.004) and a comorbid DM (β = -0.163, *p* = 0.001) were significantly associated with lower scores in the PCS.

## Discussion

In this study we retrospectively analyzed clinical data and psychological self-assessment (PHQ-9, GAD-7, SF-36) of *n* = 511 cardiac patients at Heidelberg University Hospital in Germany. We observed an overall impaired mental and physical quality of life compared to the general population, while physical quality of life was particularly reduced. Interestingly, elevated symptoms of depression were significantly associated with a former or current diagnosis of CD , an ICD or a comorbid DM rather than with HF severity per se. Further, the presence of an ICD was not only associated with higher levels of depression, but also with higher levels of anxiety. These data emphasize the need for routine psychosocial screening in this vulnerable population, particularly in ICD patients or patients with hospitalization due to CD , both of which showed a mean PHQ-9 score of > 10.

We observed an overall elevated psychosocial burden and impaired health related quality of life in all applied self-assessments compared to the general population. A mean PHQ-9 of 7.35 ± 6.09 is significantly higher than the average score of 2.69 from a large study of over 2500 people of the general German population [23]. While average GAD-7 scores range from 3 to 4 depending on the study [25, 29, 30], our data reveals a mean of 5.15 ± 5.28. With respect to HRQoL, the PCS and MCS were also lower in our sample, when comparing it to German normative data, in which a mean PCS of 51.4 and a mean MCS of 49.3 were reported [31]. This aligns with previous findings showing significantly impaired psychosocial and HRQoL in cardiac patients [32]. When comparing different subgroups (Table 2) regarding mental aspects (MCS) of HRQoL scores were notably lower after CD, with a CM, and an ICD, while regarding physical aspects (PCS) a CD or a comorbid DM were associated with lower quality of life. When controlling for other factors in a regression analysis (Table 3), CD and DM were significant predictors for lower PCS, reflecting a high somatic burden in patients with these conditions.

We observed younger age and female sex to be significantly associated with higher PHQ-9- and GAD-7-, as well as with lower HRQoL scores. In line with our findings, younger age, female sex, social isolation, having no partner [33], current smoking and alcoholism [13, 34–36] have been described as risk factors for enhanced depressive and anxious symptoms in HF patients. Similarly, lower HRQoL has been associated with female sex, younger age, higher NYHA functional class [37] and the presence of HFpEF [38, 39]. Interestingly, a former or current diagnosis of CD was markedly associated with higher PHQ-9 scores. In line with this finding decompensated HF is accompanied by high impairments of physical functioning [40] and physical limitations as well as the need for hospitalization are both associated with higher depressive and anxious symptoms [6, 40]. Although cognitive impairments are also known as a common occurrence in acutely decompensated HF [41] there is a lack of research specifically comparing depression and anxiety in acute decompensated HF to more stable HF patients. Prospective studies have shown that the prevalence of depression during acute hospitalization for myocardial infarction is up to three times higher, with approximately 20% of patients exhibiting clinically relevant depressive symptoms, of whom 32% present with moderate to severe depression [42]. Taken together, the results of our study indicate, that acute events, such as CD , may present a key event towards exacerbated depressive symptoms, warranting close monitoring.

An ICD was significantly associated with higher depression and anxiety scores. This finding aligns with previous data, which suggests that the prevalence of both depression and anxiety in ICD patients is up to four times higher than in the normal population [13, 14, 43]. Psychological models such as learned helplessness and the classic conditioning model in response to ICD shocks have been proposed to explain this association [44–46]. However, a systematic review and meta-analyses of Oshvandi et al. reported a prevalence of depression of 23.58% in ICD patients, which is significantly higher than in the general population, yet comparable to rates in other chronically ill patients [14, 43]. Beyond psychological mechanisms directly related to the implanted device, further complications associated with ICD implantation, including infection risk, device related complications and prolonged hospitalizations, may additionally contribute to patient’s psychological stress. Preventing such complications, for example through gentamicin-impregnated collagen sponges, may reduce additional psychological distress in ICD patients [47, 48]. These findings highlight the need for further research to clarify if the increased psychological burden of individuals with an ICD is attributable to the presence of the device itself or rather a reflection of the mental health burden in chronically ill patients such as those with severe HF.

Also, patients suffering from a comorbid DM reported significantly worse PHQ-9 and PCS scores, consistent with existing research indicating an increased risk for depression among patients with DM [15, 16]. Interestingly, comorbid depression in patients with DM has also been associated with an increased risk of developing HF [49, 50]. Stress-induced chronic hypercortisolism and chronic low grade inflammation may promote insulin resistance [51], enhancing overlapping pathological mechanisms similar to those in depressed HF patients.

Patients with severe HF and an LVEF < 30% presented higher PHQ-9 scores compared to all other patients. However, we observed no significant association between HF severity and depression after adjusting for other confounders in multivariate regression analyses.

In this regard, the literature shows inconsistent findings regarding the correlation between severity of HF and the extent of depression. A higher New York Heart Association (NYHA) class is consistently associated with a higher risk of depressive and anxious symptoms [13, 33, 52, 53]. However, since the NYHA classification relies on a subjective assessment of HF severity, it fails to clearly separate the somatic from the psychological. While some studies indicate that a decreased LVEF is associated with higher depressive and anxious burden [40, 54], others find no significant correlation [55] or even suggest discrepant results with increased symptoms of depression in HFpEF [39, 40, 52]. These inconsistencies may be explained through the conceptual distinction between an acute destabilization (CD), the chronic functional severity (LVEF) and functional severity (NYHA classes). While LVEF alone is a limited marker of clinical severity, NYHA class captures the functional severity more broadly. However, CD may act as a psychological stressor independently of the degree of systolic dysfunction through acute destabilization, hospitalization and uncertainty about prognosis. As NYHA classification was not assessed in this study, this dimension of HF severity may be underestimated and presents a limitation. We suggest that additional clinical factors that often go along with advanced HF - such as a CD, the presence of an ICD or a comorbid DM may also impact mental health and not just impairment of EF alone.

This study underlines the complex and multifactorial association between cardiac diseases and the mind axis. To understand this relationship the pathophysiology needs to be acknowledged. Studies have shown, that an impaired cardiac function results in an overactivity of the sympathetic nervous system [6, 56–58]. In psychological distress the body also responds with an autonomic overstimulation, which can synergize with the sympathetic overreaction in HF and result in a maladaptive process worsening cardiac function [56]. Various cross-sectional studies have found that a dysregulated inflammatory system with elevated inflammatory markers (for example C-reactive protein, interleukin 6, tumor necrosis factor alpha, and many more) also play a role in the association of cardiac diseases with depression [6, 13, 56, 57]. Further cognitive processes such as negative thinking patterns, an awareness of limited life expectancy and self-damaging behavior also contribute to depression or anxiety in HF patients [56, 57]. Psychological distress can also lead to behavioral alterations increasing the progression of cardiac diseases, through reduced physical activity [58], and lack of compliance [6, 13, 56, 57]. Also, patients with heart diseases are prone to suffer from social isolation due to their physical limitations, which may independently aggravate clinical outcomes [56]. Since impaired cardiac function is known to intensify lower HRQoL and psychological stress, this may exacerbate the aforementioned cyclical risk-relationship [59]. The potential impact of biological, cognitive, and social factors illustrates how complex the link between depression, anxiety, and cardiac disease is, and why comorbidities and their determinants cannot easily be attributed to single diagnostic categories, highlighting the need for further research into this multifaceted pathophysiology.

Since psychological impairments have been consistently associated with impaired cardiac prognosis, focusing on a targeted screening of patients with specific comorbidities that have an increased vulnerability for mental health problems (presence of an ICD, acute or past CD or patients with comorbid DM), would enable an earlier management of psychological comorbidities. This is fundamental not only for the patient’s mental wellbeing but could impact the progression of HF itself. A metanalysis published in the Lancet Psychiatry in January 2024, highlights the importance of preventive psychological interventions by showing a reduction in the incidence of major depressive disorder by 42% within 6 months and 33% within 12 months, especially in individuals with moderate to moderately severe depressive or anxiety symptoms (PHQ-9 or GAD-7 ≥ 10) [60]. Consistent with other studies [61], this underlines the effectiveness of psychosocial prevention programs especially for individuals with subclinical depression. However, guidelines don’t offer clear recommendations or a systematic screening system. The American Heart Associations (AHA) “Guidelines for Management of Heart Failure” recognize depression and anxiety as important factors in HF patients, but don’t list them as standard non-cardiac comorbidities. Depression screening is recommended, while anxiety is mainly noted in palliative care patients [62, 63]. The ESC gives more exact guidelines suggesting the screening of patients with a new diagnosis or an acute event of a cardiovascular disease, for which the short PHQ-2 and GAD-2 versions should be used [64]. Especially for HF patients with psychiatric comorbidities, a multiprofessional disease management program, containing patient education, psychosocial support, and individual cardiac treatment is crucial [65]. A metanalysis has shown that depressed HF patients undergoing cognitive behavioral therapy (CBT) showed small but significant benefits regarding depressive symptoms and quality of life [56, 66]. Regarding psychopharmaceuticals, serotonin reuptake inhibitors (SSRIs) are often recommended for patients with HF, however side effects such as QTc elongation as well as an increased bleeding risk due to an interaction with anticoagulant medication need to be considered [64]. However, research is also inconsistent due to a reported increase of all-cause mortality due to its side effects [56, 67, 68]. So far, the strongest evidence for the treatment of comorbid depression in cardiac patients exists for exercise and CBT.

Several limitations must be considered in this study. As a single-center, retrospective analysis, no causal conclusions can be drawn. Additionally, data were collected over an 11-year period (2009–2020), during which treatment standards and care practices changed. This may have introduced confounding effects that could not be fully controlled for. Given the cross-sectional design of the study, the temporal relationship between psychological symptoms and clinical factors remains unclear, underlining the need for prospective research. Missing data due to incomplete questionnaire responses may result in selection bias. In total 26% (*n* = 133) of patients had missing SF-36 data. Patients with missing SF-36 scores were significantly older than those with complete data. With no other significant differences in key clinical characteristics, most likely the impact on the overall results is expected to be limited.

Also, psychological symptoms in cardiac patients are influenced by a complex interplay of biological, psychological, and social factors, many of which were not included in this study. This complexity is reflected in the relatively low adjusted R² values of the multivariate regression models for the PHQ-9 and the GAD-7, indicating that while the models were statistically significant, the included clinical variables explain only a small proportion of the variance in depressive and anxious symptoms. Psychosocial factors such as social support, socioeconomic status, and prior psychiatric history were not captured in the present study, yet they have been shown to contribute to psychological burden in cardiac patients and may account for much of the relatively low adjusted R² values. It should also be noted that the study population presents a cardiology in-patient sample. Given the overlap of physical symptoms of e.g. HF and anxiety (e.g. dyspnea, fatigue), differentiating somatic from psychological symptoms can be challenging, potentially leading to e.g. an overestimation of anxiety or depression scoring in cardiac patients [57]. Furthermore, the HF classification based on the LVEF deviates from the current ESC guidelines, as a substantial portion of the data was collected prior to its establishment in 2016, and to allow a more precise differentiation within the HFrEF patients. However, this may limit direct comparability with other studies. Therefore, the results should be interpreted carefully, and future studies with multicentric prospective designs are needed, involving longitudinal tracking of psychological development during key clinical events such as an ICD-implantation or hospitalization due to CD.

## Conclusions

In summary, patients with a former or current CD, an ICD, or comorbid DM demonstrated significantly increased self-reported depressive symptoms, while anxious symptoms were particularly pronounced in individuals with an ICD. These findings support the recommendation for psychosocial screening of cardiac patients, particularly following a new cardiac diagnosis or acute events. Further, implementing more frequent and targeted routine screening in patients with an ICD, or a comorbid DM, rather than focusing only on specific cardiac diagnoses, may facilitate earlier identification of mental health issues even in complex cardiac patients.

## Data Availability

Data will be made available upon reasonable request.

## Abbreviations

CHD: coronary heart disease
CD: cardiac decompensation
CM: cardiomyopathy
CHD: coronary heart disease
COPD: chronic obstructive pulmonary disease
DM: diabetes mellitus
EF: Ejection fraction
GAD-7: 7-item Generalized Anxiety Disease Scale
HRQoL: health-related quality of life
HF: heart failure
HFpEF: heart failure with preserved ejection fraction
HFrEF: heart failure with reduced ejection fraction
ICD: implanted cardioverter-defibrillator
LVEF: Left ventricular ejection fraction
MCS: mental component summary scale
NYHA: New York Heart Association
PHQ-D: patient health questionnaire
PHQ-9: patient health questionnaire-9-item depression module
PCS: physical component summary scale
SF-36: short form-36 health survey
